# Can Hemogram Parameters and Derived Ratios Predict Conversion From Laparoscopic to Open Cholecystectomy?

**DOI:** 10.7759/cureus.68290

**Published:** 2024-08-31

**Authors:** Mehmet Alperen Avci, Can Akgün, Omer Buk, Dilara Karadan

**Affiliations:** 1 General Surgery, Samsun University, Samsun, TUR; 2 General Surgery, Samsun Research and Training Hospital, Samsun, TUR

**Keywords:** elective cholecystectomy, emergency cholecystectomy, predictive risk factors, laparoscopic cholecystectomy, conversion cholecystectomy

## Abstract

Backgrounds

Laparoscopic cholecystectomy (LC) is the gold standard for surgical removal of gallbladder today. In challenging cholecystectomy cases, conversion to an open technique may be necessary. Therefore, the preoperative prediction of conversion to open technique holds significant importance for patient safety and surgical strategy. In the literature, conversion to open cholecystectomy has been associated with many contradictory predictive factors. The aim of this study is to identify and comprehensively evaluate the predictive laboratory parameters and ratios associated with the conversion from laparoscopic to open cholecystectomy.

Methods

In this historical cohort study, the data of patients who were scheduled for and underwent LC between January 1, 2018, and September 1, 2023, were retrospectively evaluated. The preoperative laboratory findings and surgical notes of the patients were reviewed retrospectively from the archives. The correlation between patient data and the cholecystectomy groups was analyzed, and comparisons were made between the groups.

Results

All 160 patients initially underwent a laparoscopic approach. In emergency cases, a statistically significant association was found between lymphocyte count (p = 0.017) and lymphocyte-to-monocyte ratio (LMR) (p = 0.041) with operations completed laparoscopically and between neutrophil-to-lymphocyte ratio (NLR) (p = 0.007) and Systemic Inflammatory Response Index (SIRI) (p = 0.031) with operations converted to open surgery. In elective cases, gamma-glutamyl transferase (GGT) (p = 0.024) and total bilirubin (TBIL) (p = 0.003) were found to have a statistically significant association with operations converted to open surgery. In the logistic regression analysis, hematological parameters and ratios were not found to have a statistically significant relationship in predicting the conversion to open surgery.

Conclusion

Although significant differences were observed in laboratory parameters and derived ratios such as the NLR and LMR, logistic regression analysis did not identify any of these measures as significant predictors of conversion from laparoscopic to open surgery. Further prospective studies with larger sample sizes are needed in this area.

## Introduction

Laparoscopic cholecystectomy (LC) has become the most commonly used and gold-standard surgical approach for symptomatic cholelithiasis nowadays [1,2]. The superiority of this approach over the open cholecystectomy technique can be attributed to the lower overall complication rate and shorter hospital stay [3,4]. In challenging cholecystectomy cases, surgeons may need to transition from laparoscopic to open technique. Reviewing the literature, peritoneal adhesions and gallbladder inflammation have been frequently implicated in the conversion from laparoscopic to open cholecystectomy, with conversion rates reported between 2% and 15% [5-7]. Upon examination of conversion cases, a high incidence of infection, postoperative complications, need for additional procedures, frequent readmissions, and prolonged hospital stays have been observed [8-10]. Therefore, preoperative prediction of the risk of conversion from laparoscopic to open cholecystectomy is crucial for patient safety, treatment cost, and surgical strategy determination. A review of the literature has shown that in the conversion from laparoscopic to open cholecystectomy, various inconsistent laboratory biomarkers are predicted as significant predictive factors. Parameters derived from complete blood counts, such as neutrophil-to-lymphocyte ratio (NLR), platelet-to-lymphocyte ratio (PLR), Systemic Inflammatory Index (SII), and Systemic Inflammatory Response Index (SIRI), are typically employed to predict acute and chronic inflammation. Additionally, the literature indicates that these markers are used for various purposes in LC, including predicting postoperative pain and the likelihood of conversion to open surgery [7,11-13].

The aim of this study is to investigate whether specific hemogram-derived ratios and indices can reliably predict the necessity for conversion to open surgery in patients undergoing LC. This investigation encompasses both emergency and elective surgical cases. By comparing those who required conversion to open surgery with those who successfully completed the laparoscopic procedure, the study seeks to determine the predictive value of these hematological markers in foreseeing surgical outcomes.

## Materials and methods

Study design

This study was designed as a retrospective analysis and was conducted following the approval of the local ethics committee. The ethical approval for the study was obtained from the Samsun University Clinical Research Ethics Committee on January 31, 2024, with the protocol number 2024/3/2. The research was carried out at the General Surgery Clinic of Samsun University Training and Research Hospital, Samsun, Turkey focusing on patients who were scheduled for LC between January 1, 2018, and September 1, 2023. The study included cases where the initial laparoscopic procedure had to be converted to open surgery due to intraoperative complications or other unforeseen factors, as well as those where the procedure was started and successfully completed laparoscopically.

Study population

The study population comprised patients aged 18 to 90 years who underwent cholecystectomy due to symptomatic gallbladder disease. To maintain the integrity of the study, only those with no prior history of abdominal surgery or significant comorbid conditions were included. This exclusion criterion was essential to avoid confounding factors that could potentially influence surgical outcomes or laboratory parameters.

Preoperative laboratory data were meticulously reviewed, with the most recent results obtained within 24 hours prior to the surgery being included in the analysis. Patients whose preoperative lab results were older than 24 hours were excluded, ensuring that the data reflected the patients’ most current clinical status. Patients with acute or chronic renal failure, significant liver function abnormalities (defined as liver function test results three times or more above the upper limit of normal), or an international normalized ratio (INR) above 1.5 were excluded from the evaluation. These thresholds were established based on previous studies, considering their potential to contribute to chronic inflammation or perioperative complications.

Grouping and matching

Patients were divided into two groups based on the urgency of the surgical procedure: the emergency group and the elective group. To enhance the reliability of the comparative analysis, a 1:1 matching was performed between the two groups. Matching criteria included age, gender, body mass index (BMI), ethnicity, and underlying pathological conditions. This careful matching process was designed to minimize bias and ensure that the two groups were comparable in terms of key demographic and clinical variables.

Within each of the emergency and elective groups, consistency was maintained by ensuring that patients were compared only with others within the same group. The final sample size was determined based on the number of cases in each group that required conversion from laparoscopic to open surgery. It was planned that each group would consist of 40 patients who underwent LC and 40 patients who required conversion to open surgery. This approach allowed for a balanced comparison of outcomes across different surgical scenarios.

Data collection and analysis

A comprehensive data collection process was undertaken, during which the preoperative serum biochemistry and hemogram parameters of all patients were systematically recorded. In addition to standard hemogram parameters, several derived ratios were calculated to provide deeper insights into the patients’ inflammatory and immune status. These ratios included the NLR, neutrophil-to-monocyte ratio (NMR), lymphocyte-to-monocyte ratio (LMR), and the SIRI, calculated as (neutrophil*monocyte/lymphocyte).

The inclusion of these derived ratios aimed to enhance the predictive power of the study, as these markers have been increasingly recognized for their role in assessing the inflammatory response and potential surgical outcomes. By incorporating both standard and derived parameters, the study sought to provide a comprehensive evaluation of the factors that may influence the need for conversion from laparoscopic to open cholecystectomy.

Overall, this methodological approach was designed to ensure rigorous data collection, careful patient matching, and thorough analysis, thereby contributing to a robust and reliable study outcome.

The primary outcome

The primary outcome of this study is to evaluate the predictive value of specific hemogram-derived ratios and indices in determining the likelihood of conversion from laparoscopic to open cholecystectomy. These ratios include the NLR, NMR, LMR, and SIRI. By analyzing these parameters, the study aims to identify whether they can serve as reliable predictors of the need for open surgery in both emergency and elective laparoscopic procedures.

Statistical analyses

Statistical analysis was performed using dedicated statistical software (IBM SPSS Statistics 22, SPSS Inc., an IBM Co., Somers, NY). The descriptive statistics were conducted to provide information about the general characteristics of the study groups. Data for quantitative measurements were presented as mean and standard deviation (mean ± SD), while data for qualitative variables were described using counts (n) and percentages (%). Group differences for quantitative measurements were assessed using independent samples T-test, while differences for qualitative variables among groups were evaluated using the Chi-square test. We also conducted a logistic regression analysis to identify factors that predict the conversion to open surgery. For statistical significance, a p-value of less than 0.05 was considered.

## Results

**Figure 1:**
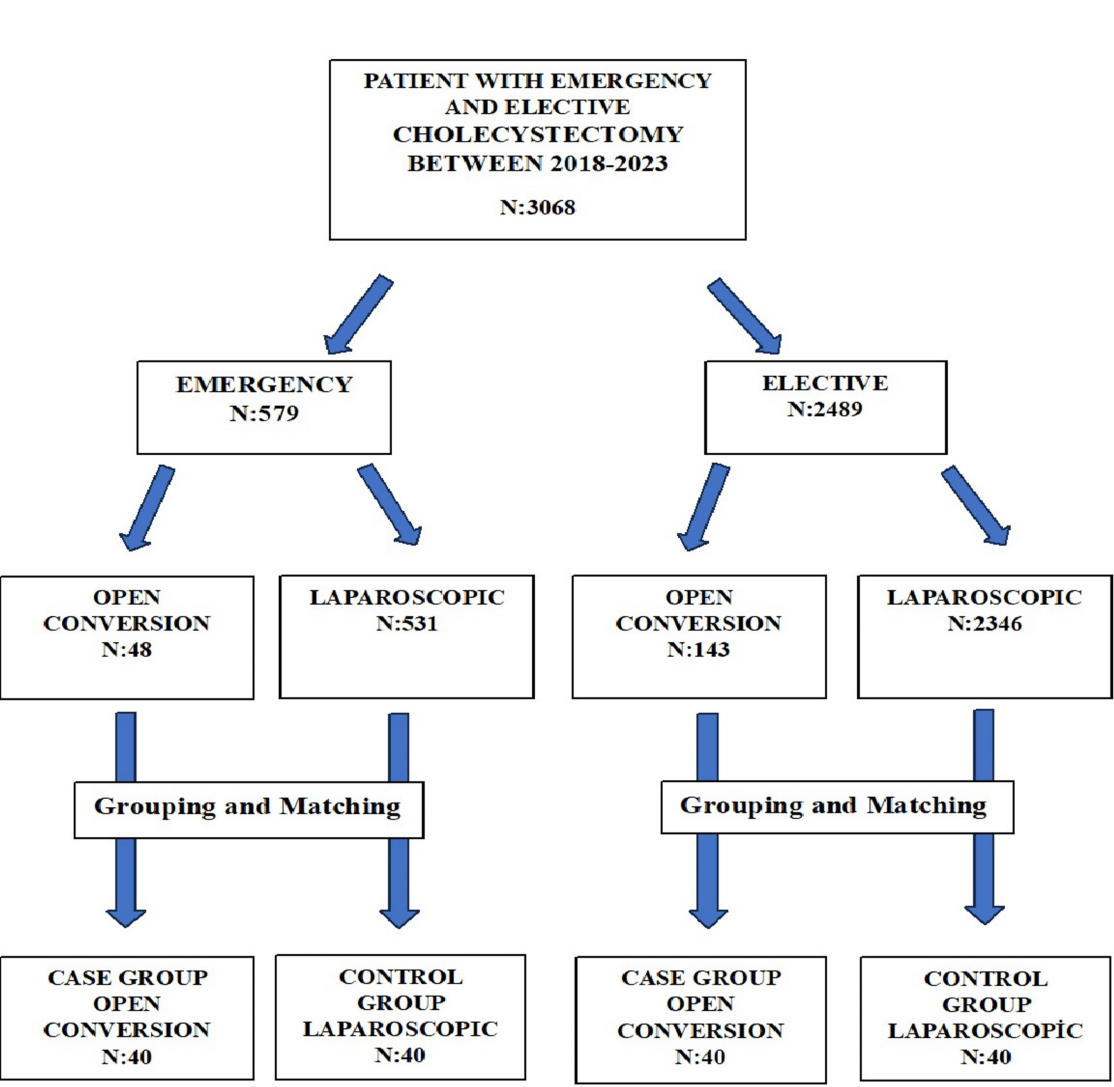
Laparoscopic and open conversion grouping and matching diagram

Between January 1, 2018, and September 1, 2023, a total of 3,068 patients underwent cholecystectomy due to symptomatic gallbladder complaints. Among these patients, 579 were operated on an emergency basis, and 2,489 underwent elective surgery. Of the patients who underwent emergency surgery, 48 were converted from laparoscopic to open surgery, while 531 were completed laparoscopically. Among the patients who underwent elective surgery, 143 required conversion from laparoscopic to open surgery, and 2,346 were completed laparoscopically (Figure 1).

Emergency cases

Table 1 displays a comparison of various parameters between patients who completed laparoscopic surgery and those who required conversion to open surgery in emergency cases. The mean operation time for patients converted to open surgery was longer (107.60 minutes) compared to those who completed laparoscopic surgery (96.10 minutes), though this difference was not statistically significant (p=0.099). Among the laboratory parameters, NLR (p = 0.007) was found to be significantly higher in patients requiring conversion to open surgery. Additionally, lymphocyte count, LMR, and SIRI also showed significant differences (p = 0.017, p = 0.041, and p = 0.031, respectively). No significant differences were observed in other parameters such as AST, ALT, ALP, gamma-glutamyl transferase (GGT), and total bilirubin (TBIL).

**Table 1:** Comparison of data between patients who completed laparoscopic surgery and those who required conversion to open surgery in emergency cases TBIL, total bilirubin; DBIL, direct bilirubin; GGT, gamma-glutamyl transferase; SIRI, Systemic Inflammatory Response Index; NLR, neutrophil-to-lymphocyte ratio; LMR, lymphocyte-to-monocyte ratio; NMR, neutrophil-to-monocyte ratio

Parameter	Open surgery (N = 40)	Laparoscopic surgery (N = 40)	P-value
Operation duration (minute)	45.00 - 204.00 (107.60 ± 39.29)	45.00 - 189.00 (96.10 ± 34.06)	0.099
AST	7.70 - 142.60 (33.23 ± 28.84)	9.70 - 205.00 (36.73 ± 39.15)	0.479
ALT	5.00 - 167.00 (30.83 ± 31.90)	6.00 - 179.00 (41.11 ± 39.02)	0.110
ALP	23.00 - 987.00 (121.15 ± 66.44)	23.00 - 302.10 (92.81 ± 59.96)	0.606
GGT	16.00 - 934.00 (120.23 ± 01.25)	10.00 - 882.00 (123.25 ± 163.52)	0.373
TBIL	0.13 - 13.20 (1.27 ± 2.11)	0.20 - 5.69 (0.96 ± 0.98)	0.441
DBIL	0.01 - 8.10 (0.45 ± 1.28)	0.08 - 2.58 (0.31 ± 0.45)	0.795
Amylase	16.00 - 154.00 (59.90 ± 25.71)	19.00 - 123.00 (65.28 ± 26.13)	0.266
WBC	4.23 - 21.00 (8.62 ± 3.43)	3.8 - 13.58 (7.90 ± 2.55)	0.587
Neutrophils	1.91 - 18.50 (5.98 ± 3.26)	1.90 - 9.60 (4.93 ± 1.93)	0.240
Lymphocytes	0.40 - 3.95 (1.75 ± 0.86)	0.97 - 4.08 (2.12 ± 0.69)	0.017
Monocytes	0.10 - 6.40 (0.81 ± 1.01)	0.30 - 1.24 (0.61 ± 0.23)	0.783
NLR	0.69 - 12.54 (4.38 ± 3.14)	1.11 - 6.03 (2.45 ± 1.07)	0.007
NMR	0.59 - 36.00 (11.13 ± 8.22)	3.80 - 15.39 (8.36 ± 2.73)	0.341
LMR	0.44 - 11.00 (3.22 ± 2.17)	2.01 - 7.33 (3.73 ± 1.41)	0.041
SIRI	0.33 - 9.05 (3.08 ± 2.61)	0.41 - 3.64 (1.53 ± 0.87)	0.031

Elective cases

Table 2 summarizes the data comparison for elective cases. GGT and TBIL also demonstrated significant differences (p=0.024 and p=0.003, respectively). The other parameters, including WBC, neutrophil, lymphocyte, monocyte, NLR, NMR, LMR, and SIRI, did not show significant differences between the groups (p>0.05).

**Table 2:** Comparison of data between patients who completed laparoscopic surgery and those who required conversion to open surgery in elective cases TBIL, total bilirubin; DBIL, direct bilirubin; GGT, gamma-glutamyl transferase; SIRI, Systemic Inflammatory Response Index; NLR, neutrophil-to-lymphocyte ratio; LMR, lymphocyte-to-monocyte ratio; NMR, neutrophil-to-monocyte ratio

Parameter	Open surgery (N = 40)	Laparoscopic surgery (N = 40)	P-value
Operation duration (minute)	40.00 - 210.00 (92.93 ± 44.58)	40.00 - 140.00 (65.55 ± 18.89)	0.008
AST	10.90 - 41.00 (23.63 ± 6.68)	10.00 - 75.00 (24.40 ± 13.06)	0.410
ALT	8.00 - 86.00 (23.66 ± 13.58)	7.00 - 71.00 (23.54 ± 16.13)	0.307
ALP	34.00 - 610.00 (100.08 ± 90.72)	23.00 - 138.00 (75.41 ± 25.86)	0.207
GGT	7.00 - 647.00 (87.78 ± 124.34)	9.00 - 246.00 (43.58 ± 46.31)	0.024
TBIL	0.27 - 2.30 (0.82 ± 0.40)	0.18 - 2.02 (0.62 ± 0.34)	0.003
DBIL	0.05 - 0.70 (0.18 ± 0.15)	0.05 - 1.00 (0.16 ± 0.15)	0.213
Amylase	34.00 - 135.00 (66.39 ± 24.43)	22.90 - 146.00 (66.98 ± 26.59)	0.996
WBC	3.50 - 12.50 (6.88 ± 1.90)	4.49 - 13.09 (7.71 ± 2.22)	0.130
Neutrophils	2.10 - 8.80 (4.16 ± 1.43)	2.29 - 9.86 (4.66 ± 1.57)	0.130
Lymphocytes	1.00 - 3.30 (1.99 ± 0.60)	1.03 - 4.40 (2.23 ± 0.75)	0.218
Monocytes	0.30 - 0.98 (0.53 ± 0.16)	0.20 - 0.95 (0.56 ± 0.18)	0.328
NLR	1.15 - 4.83 (2.20 ± 0.80)	1.04 - 4.23 (2.18 ± 0.67)	0.851
NMR	4.72 - 19.33 (8.17 ± 2.73)	3.05 - 17.00 (8.74 ± 2.71)	0.157
LMR	2.17 - 6.60 (3.88 ± 1.05)	1.21 - 10.00 (4.23 ± 1.50)	0.223
SIRI	0.57 - 2.63 (1.16 ± 0.55)	0.34 - 3.22 (1.24 ± 0.59)	0.341

Table 3 presents the results of logistic regression analyses for predicting conversion to open surgery. In emergency cases and elective cases, any hemogram parameters or ratios did not reach statistical significance in this context.

**Table 3:** Logistic regression analysis of hemogram parameters and derived ratios for predicting conversion to open surgery SIRI, Systemic Inflammatory Response Index; NLR, neutrophil-to-lymphocyte ratio; LMR, lymphocyte-to-monocyte ratio; NMR, neutrophil-to-monocyte ratio

Variable	B	SE	Wald	df	Sig.	Exp(B)	95% CI for Exp(B)	
							Lower	Upper
WBC	-0.520	1.394	0.139	1	0.709	0.594	0.039	9.132
Neutrophils	2.102	1.607	1.710	1	0.191	8.184	0.351	191.111
Lymphocytes	-1.536	2.586	0.353	1	0.553	0.215	0.001	34.246
Monocytes	3.368	3.399	0.982	1	0.322	29.015	0.037	22702.723
NLR	0.399	1.355	0.087	1	0.768	1.490	0.105	21.206
NMR	-0.527	0.528	0.998	1	0.318	0.590	0.210	1.661
LMR	0.336	0.921	0.133	1	0.715	1.400	0.230	8.503
SIRI	-3.167	2.280	1.930	1	0.165	0.042	0.000	3.673
Constant	2.602	2.840	0.839	1	0.360	13.487		

## Discussion

In our study, it was found that in emergency cases, lymphocyte count and LMR were significantly higher in operations completed laparoscopically, whereas NLR and SIRI were significantly elevated in operations that were converted to open surgery. In elective cases, GGT and TBIL were significantly higher in operations that were converted to open surgery. However, in both elective and emergency operations, hematological parameters and ratios were not found to be statistically significant in predicting the conversion from laparoscopic to open surgery in logistic regression analysis.

With the advancement of technology, laparoscopic surgery has become the gold standard in surgical practice worldwide due to its minimally invasive nature, safety, lower rates of postoperative complications, faster recovery process, and shorter hospital stays. Nevertheless, in some challenging cholecystectomy cases, it may be necessary to convert from the planned laparoscopic technique to open surgery to ensure patient safety and prevent complications [14,15]. Particularly in cases involving adhesions secondary to inflammation and acute cholecystitis, several studies have demonstrated a higher rate of conversion to open surgery [16,17]. Among hematological parameters, WBC is utilized to assess the severity of inflammation, as outlined in the Tokyo (2018) guidelines [18]. Additionally, ratios of hematological parameters, such as NLR, LMR, NMR, and SIRI, are rapidly accessible markers used to evaluate the inflammatory response. These ratios provide critical insights into the patient's inflammatory activity [19-21].

In some studies, NLR, NMR, PLR, and SIRI values have been shown to have a significant association with difficult cholecystectomy [22-24]. Moloney et al. even reported that in elective LC cases, an NLR value greater than three was associated with a higher risk of conversion to open surgery [20]. Conversely, other studies have found no significant relationship between NLR and conversion from LC to open surgery [25,26]. A review of the literature reveals that elevated levels of biochemical biomarkers such as AST, ALT, ALP, GGT, and bilirubin are significantly associated with conversion cholecystectomy [27,28]. However, in one study, no significant relationship was found between ALP, ALT, and bilirubin levels and conversion cholecystectomy [29]. In our study, NLR and SIRI values in emergency cases and GGT and TBIL values in elective cases were found to be significantly associated with conversion to open surgery. However, our logistic regression analysis did not reveal a significant relationship between the hematological parameters and ratios evaluated and the conversion to open surgery.

This study has several limitations that must be acknowledged. First, the retrospective design of this study inherently limits the ability to establish causality and may introduce bias. Unlike a prospective study, which could offer more controlled and systematic data collection, our retrospective approach relies on existing records, which may not capture all relevant variables. Additionally, we did not include all patients but rather selected those meeting specific criteria, which could impact the generalizability of the findings.

Moreover, patients with chronic kidney disease and those with severe chronic inflammation were excluded from the study, as these conditions could significantly affect hemogram parameters and skew the results. We also did not stratify patients based on smoking status or obesity, both of which are known to influence hemogram indices. These factors could have provided further insights or altered the observed associations. Addressing these limitations in future research could enhance the robustness and applicability of the findings.

## Conclusions

This study assessed the predictive value of hemogram parameters and derived ratios for conversion from laparoscopic to open cholecystectomy. Although differences in parameters like the NLR and LMR were noted, logistic regression did not identify any of these measures as significant predictors of conversion. This indicates that while preliminary analyses show differences, these indices do not reliably predict conversion in either urgent or elective cases. Further prospective studies with larger samples are needed to clarify their potential role and to identify better predictors for preoperative risk assessment.
